# Typing Discrepancy Between Phenotypic and Molecular Characterization Revealing an Emerging Biovar 9 Variant of Smooth Phage-Resistant *B. abortus* Strain 8416 in China

**DOI:** 10.3389/fmicb.2015.01375

**Published:** 2015-12-08

**Authors:** Yao-Xia Kang, Xu-Ming Li, Dong-Ri Piao, Guo-Zhong Tian, Hai Jiang, En-Hou Jia, Liang Lin, Bu-Yun Cui, Yung-Fu Chang, Xiao-Kui Guo, Yong-Zhang Zhu

**Affiliations:** ^1^Department of Immunology and Microbiology, Institutes of Medical Sciences, Shanghai Jiao Tong University School of MedicineShanghai, China; ^2^Baotou Municipal Center for Disease Control and PreventionBaotou, China; ^3^State Key Laboratory for Infectious Disease Prevention and Control, Collaborative Innovation Center for Diagnosis and Treatment of Infectious Diseases, National Institute for Communicable Disease Control and PreventionBeijing, China; ^4^Department of Population Medicine and Diagnostic Sciences, College of Veterinary Medicine, Cornell University, IthacaNY, USA

**Keywords:** *B. abortus*, smooth phage-resistant (SPR), MLVA typing, unusual biochemical reactions

## Abstract

A newly isolated smooth colony morphology phage-resistant strain 8416 isolated from a 45-year-old cattle farm cleaner with clinical features of brucellosis in China was reported. The most unusual phenotype was its resistance to two *Brucella* phages Tbilisi and Weybridge, but sensitive to Berkeley 2, a pattern similar to that of *Brucella melitensis* biovar 1. VITEK 2 biochemical identification system found that both strain 8416 and *B. melitensis* strains shared positive ILATk, but negative in other *B. abortus* strains. However, routine biochemical and phenotypic characteristics of strain 8416 were most similar to that of *B. abortus* biovar 9 except CO_2_ requirement. In addition, multiple PCR molecular typing assays including AMOS-PCR, *B. abortus* special PCR (B-ab PCR) and a novel sub-biovar typing PCR, indicated that strain 8416 may belong to either biovar 3b or 9 of *B. abortus*. Surprisingly, further MLVA typing results showed that strain 8416 was most closely related to *B. abortus* biovar 3 in the *Brucella* MLVA database, primarily differing in 4 out of 16 screened loci. Therefore, due to the unusual discrepancy between phenotypic (biochemical reactions and particular phage lysis profile) and molecular typing characteristics, strain 8416 could not be exactly classified to any of the existing *B. abortus* biovars and might be a new variant of *B. abortus* biovar 9. The present study also indicates that the present phage typing scheme for *Brucella sp.* is subject to variation and the routine *Brucella* biovar typing needs further studies.

Because of unstable phenotypic characteristics among *Brucella* strains, it is somewhat difficult to define atypical strains into standard biovars. For instance, the susceptibility of smooth *B. abortus* strains to lysis by most of *brucella* phages, such as Tbilisi (Tb), Firenze (Fi), Weybridge (Wb), and Berkeley 2 (BK_2_), is commonly regarded as one of the routine criteria to differentiate this organism from other *Brucella* species. However, the majority of *B. abortus* strains resistant to *Brucella* phage have been currently reported primarily due to variation from smooth to rough form during normal *in vitro* culture. Since the first smooth phage-resistant strain (SPR) of *B. abortus* isolated from bovine tissue was reported in 1973 (Corbel and Morris, 1974, 1975), a similar study describing SPR strains has not been reported yet. In this study, we report a newly isolated SPR strain, strain 8416 from a patient with brucellosis in the Inner Mongolia Autonomous Region of China on 2012. Actually, it was the only *B. abortus* strain among a total of 197 *Brucella* strains isolated and authenticated by Chinese CDC during this year. The Inner Mongolia Autonomous Region has the highest incidence, responsible for about more than 40% of reported cases in China (Zhang et al., 2010; Chen et al., 2013). Interestingly, the unique phenotypical characteristics of the *B. abortus* SPR strain 8416, determined by routine biotyping for the identification of *Brucella* species and biovars, did not completely fit into any of the recognized classification biovars, indicating the potential presence of a new variant of *B. abortus* biovar 3.

## Materials and Methods

### Bacterial Isolation and Used Strains

The protocol for this study was approved by ethics committee of local disease control and Prevention Research Center of the Inner Mongolia Autonomous Region and Baotou Municipal Center for Disease Control and Prevention. In June 2012, two workers from a cattle farm in Sichuan province, presenting fever, night sweat and soreness of waist, arthralgia and muscle weakness, were admitted to one local hospital in the Inner Mongolia Autonomous Region. The serum samples from these two patients were strongly positive to *Brucella* by both Rose-Bengal-plate-agglutination-test (RBPT) and Serum Agglutination Test (SAT) with titers of 1/320 according to standard procedures. Moreover, the two serum samples were also confirmed by positive ELISA results with *Brucella* IgG (>150 U/ml) and IgM (>60 U/ml) (*Brucella* IgG and IgM ELISA kits, IBL Germany). At the same time, the blood culture of the two patients were inoculated in a dual-phase coloration blood culture bottle (BioMerieux Inc., Durham, USA) at 37°C for 2–3 weeks at the diagnostic laboratory of Baotou Municipal Center for Disease Control and Prevention, the Inner Mongolia Autonomous Region of China. However, only one blood sample from a 45-year-old male janitor yielded a positive culture result. The isolated strain 8416 displayed smooth, tiny, white, shiny and translucent colonies on solid agar after 3 days of incubation. The strain 8416 was sub-cultured on blood plate with 5% CO_2_ and displayed typical colonies with small Gram-negative coccobacilli. The strain was sent to department of brucellosis, Chinese Communicable Disease Control and Prevention (Chinese CDC) for further analysis and identification. The reference strains including *B. abortus* biovar 1 to 7 and 9, strains: 544A (ATCC 23448), 86/8/59 (ATCC 23449), Tulya (ATCC 23450), 292 (ATCC 23451), B3196 (ATCC 23452), 870 (ATCC 23453), 63/75, and C68 (ATCC 23455), *B. melitensis* biovar 1 to 3, strains: 16M (ATCC 23456), 63/9 (ATCC 23457) and Ether (ATCC 23458)), *B. suis* biovar 1 to 5, strains: 1330S (ATCC 23444), Thomsen (ATCC 23445), 686 (ATCC 23446), 40 (ATCC 23447), and 513, *B. neotomae* RM6/66 (ATCC 23365), *B. ovis* 63/290 (ATCC 25840), and *B. canis* 5K33 (ATCC 23459) were used as controls for phenotype typing, biochemical and/or molecular analysis.

### Analysis of Phenotypic Characteristics

At first, to exclude mixed cultures of different biovars and phage carrier state, the strain used in this study was subjected to a single cloned isolation for successive three times to confirm no variable colonial morphology as described by Jones et al. (1962). The strain was further characterized by using the classical *Brucella* phenotypic identification procedures, such as CO_2_ requirement, H_2_S production, dye sensitivity by basic fuchsin and thionin, agglutination with monospecific antisera, and phage typing as described by Alton GG (Alton et al., 1975). *Brucella* monospecific antisera to A, M, and R (rough) and *Brucella* phages Tb, Wb, and Bk_2_ were used according to standard protocol of the Chinese CDC (Jiang et al., 2013) to characterize this strain. All of phenotypic characterizations in this study were repeated at least three times to make sure the results are repeatable.

### Molecular Typing Identification

*Brucella* strains were inactivated by suspending one loop from a solid bacterial culture in 200 μl DNA storage buffer. Total genomic DNA was extracted using the DNeasy Blood & Tissue Kit (Qiagen China Ltd., Beijing, China) following the manufacture’s instruction. The PCR assay targeting bcsp31, was performed to confirm the *Brucella* genus as previously described (Bounaadja et al., 2009), and species-level using the routine *Abortus-Melitensis-Ovis-Suis* PCR (AMOS-PCR) (Bricker and Halling, 1994). Furthermore, *B. abortus* B-ab PCR and a novel PCR to differentiate *B. abortus* biovar 3a, 3b, 5, 6, and 9 were performed as previously described (Ocampo-Sosa et al., 2005; Huber et al., 2009).

### Multiple Locus Variable Number Tandem Repeat Analysis (MLVA) Genotyping

Multiple locus variable number tandem repeat analysis (MLVA) was performed as previously described by Le Fleche et al. (2006) and by Jiang et al. (2013), respectively. The 16 primer pairs comprised three main groups: panel 1 including bruce06, 08, 11, 12, 42, 43, 45, and 55 for species identification, panel 2A (bruce18, 19, and 21), and panel 2B (bruce04, 07, 09, 16, and 30) for further subspecies differentiation were used.

### Biochemical Identification by VITEK 2 System

A total of 47 biochemical reactions of the *Brucella* strains were analyzed using the standard Gram-negative bacteria identification card on automatic VITEK 2 system according to the manufacturer’s instructions.

## Results

### Routine Phenotypic Typing Characteristics

**FIGURE 1:**
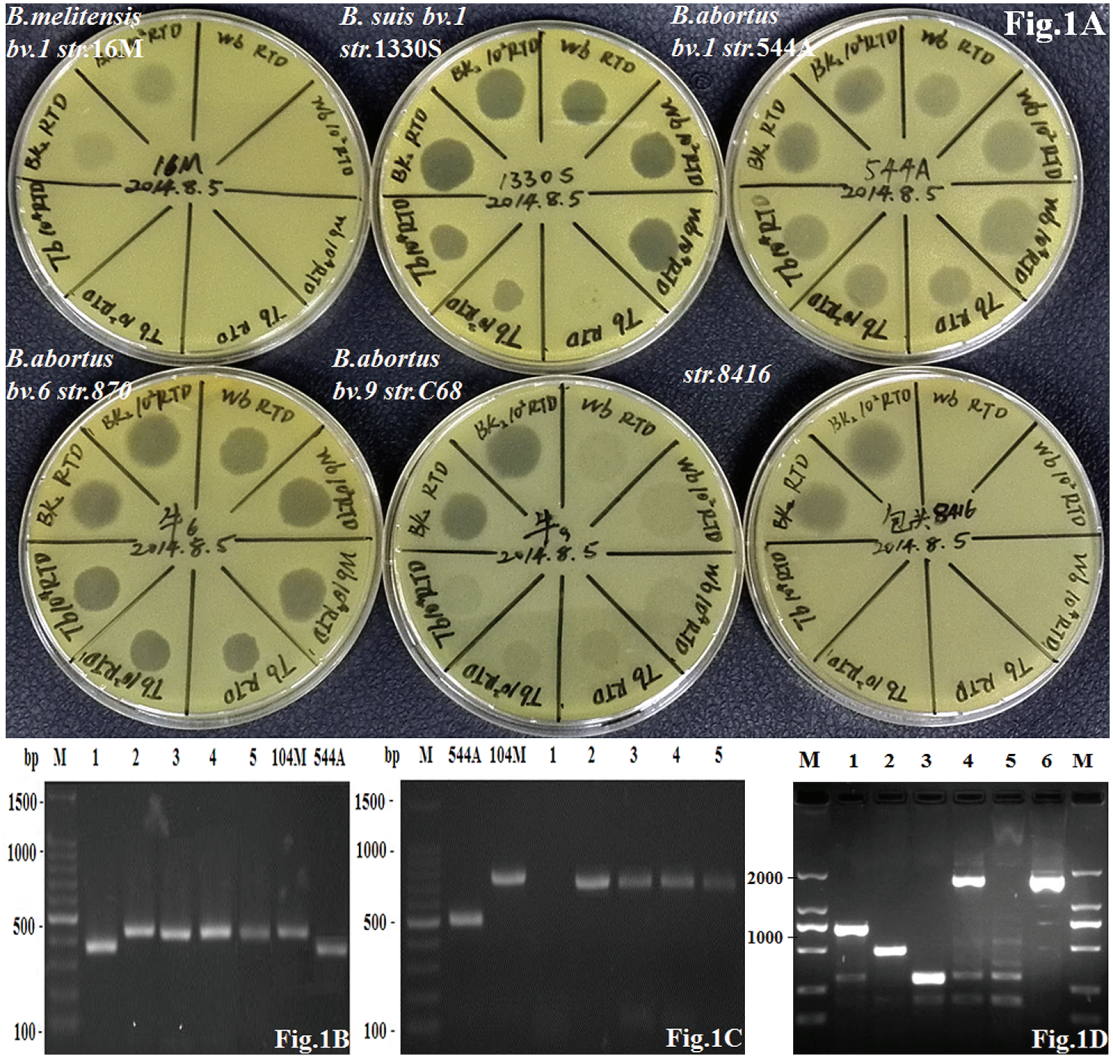
**(A)** The lysis patterns of phage Tb, Wb and Bk_2_ to *Brucella abortus* strain 8416, *B. abortus* biovar 1 strain 544A (A is indicated as *B.* ***a****bortus*), *B. melitensis* biovar 1 strain 16M (M is indicated as *B.* ***m****elitensis*), and *B. suis* biovar 1 strain 1330S (S is indicated as *B.* ***s****uis*) as well as *B. abortus* biovar 6 strain 870 and biovar 9 strain C68; **(B)** Amplification of DNA fragments from different *Brucella* strains. Genomic DNA was amplified by the B-ab PCR assay. 1: strain 8416; 2–5: four *B. melitensis* field strains; 104 M: *B. melitensis* biovar 1 strain 104M; 544A: *B. abortus* biovar 1 strain 544A; **(C)** Amplification of DNA fragments from different *Brucella* strains. Genomic DNA was amplified by AMOS-PCR assay. 1: strain 8416; 2–5: four *B. melitensis* field strains; 104 M: *B. melitensis* biovar 1 strain 104M; 544A: *B. abortus* biovar 1 strain 544A; **(D)** Amplification of DNA fragments from different *Brucella* strains. Genomic DNA was amplified by new PCR assay identifying *B. abortus* biovar 3b, 5, 6, and 9. 1: *B. melitensis* biovar 1 strain 16M; 2: *B. abortus* biovar 1 strain 544A; 3: *B. suis* biovar 1 strain 1330S; 4: *B. abortus* biovar 9 strain C68; 5: *B. abortus* biovar 3a strain Tulya; 6: strain 8416.

According to routine phenotypic analysis, strain 8416 was anti-R negative and H_2_S positive, agglutination with anti-M serum but not anti-A serum and grew in the presence of thionine and fuchsin dyes (**Table 1**). Moreover, it was not lysed by Tb and Wb phages both in 1× RTD (Routine Test Dilution) and 10^4^× RTD, but lysed by BK_2_ phage both in 1× RTD and 10^2^× RTD (**Figure 1A**). Thus, the particular phenotypic profiles of the strain 8416 were more similar to that of the classic characteristics of *B. abortus* biovars 9.

**Table 1:** Comparison of phenotypic characteristics and *Brucella* phage lysis profiles of *Brucella abortus* strain 8416 and other *Brucella* reference strains.

Strain	Growth characteristics				Mono specific phage at					Brucella MLVA16																Interpretation
				
					Sera		RTD																			
				
	CO_ 2_ requirement	H_2_S production	Thionin	Fuschin	A	M	*Tb*	*Wb*	*BK_2_*	bruce06	bruce08	bruce11	bruce12	bruce42	bruce43	bruce45	bruce55	bruce18	brucel9	bruce21	bruce04	bruce07	bruce09	bruce16	bruce30	
*8416*	±	+	+	+	–	+	–	–	+	4	5	4	12	2	2	3	2	6	42	8	6	6	7	3	3	*B. abortus* biovar 9 variant
*544A*	–	+	–	+	+	–	+	+	+	4	5	4	12	2	2	3	3	5	21	8	3	5	3	4	5	*B. abortus* biovar 1
*Tulya*	±	+	+	+	+	–	+	+	+	3	5	4	11	2	2	3	3	8	20	8	6	5	3	11	5	*B. abortus* biovar 3
*870*	–	–	+	+	+	–	+	+	+	3	3	6	5	3	3	12	3	7	42	8	3	2	2	3	3	*B. abortus* biovar 6
*C68*	–	+	+	+	–	+	+	+	+	6	3	6	5	3	3	12	3	7	42	8	3	2	2	2	3	*B. abortus* biovar 9
*16M*	–	–	+	+	+	+	–	–	+	3	4	2	13	4	2	3	3	5	18	6	2	5	8	3	6	*B. melitensis* biovar 1
*Ether*	–	–	+	+	+	+	–	–	+	7	3	5	5	12	3	13	9	7	42	8	3	1	1	3	3	*B. melitensis* biovar 3
*1330S*	–	++	+	–	+	–	–	+	+	2	3	6	10	4	1	5	2	4	19	9	6	6	5	5	3	*B. suis* biovar 1


### Biochemical Identification of Automatic VITEK 2 System

Four biochemical indicators ProA (L-pyrrolydonyl-arylamidase), TyrA (tyrose arylamidase), URE (urease), and GlyA could be used to distinguish *Brucella* species. All of eight *B. abortus* reference strains and 21 field strains were positive in ILATk (L-lactate alkalization), but it was negative in strain 8416, three *B. melitensis* reference strains and 92 field strains (Cui BuYun’s unpublished data). This result indicated that strain 8416 showed special biochemical characteristics distinct from that of *B. abortus* strains.

### Molecular Typing Identification

Strain 8416 was identified as *B. abortus* by the combination of bcsp31 PCR (223-bp, data not shown) and B-ab PCR (370-bp) (**Figure 1B**) but not as biovar 1, 2, and 4 of *B. abortus* according to AMOS-PCR (**Figure 1C**). The novel PCR assay was used to compare strain 8416 to *B. abortus* biovar 3b, 5, 6, and 9, and found that the PCR product of 1.7 kb from strain 8416 was similar to *B. abortus* biovar 3b, 5, 6, and 9, but not to other *B. abortus* biovars (**Figure 1D**).

### MLVA Genotyping

According to *Brucella* MLVA typing database (Grissa et al., 2008), 16 loci of MLVA matching results displayed that strain 8416 was closely related to *B. abortus* biovar 3 (Jiang et al., 2013), but primarily different in four variable loci, bruce04, bruce07, bruce11, and bruce55 (**Table 2**).

**Table 2:** Comparison of *Brucella* MLVA typing results of *B. abortus* strain 8416 and the most closely related *B. aborus* biovar 3 field strains in the *Brucella* MLVA database.

Strain name	Distance	BaseView	Strain	Host	Isolated_in	*Species-biovar*	Contact	Group	Year	MLVA8	MLVA11	MLVA16	Bruce06-1322	Bruce08-1134	Bruce11-211	Bruce12-73	Bruce42-424	Bruce43-379	Bruce45-233	Bruce55-2066	Bruce18-339	Bruce19-324	Bruce21-329	Bruce04-1543	Bruce07-1250	Bruce09-588	Bruce16-548	Bruce30-1505
8416			8416	Human	Inner Mongolia, China				2012			MLVA16	4	5	4	12	2	2	3	2	6	42	8	6	6	7	3	3
2013Jiang#095	0	Brucella2013		Human	Inner Mongolia, China	*B. abortus biovar 3*	Buyun Cui		2012	30		MLVA16	4	5	4	12	2	2	3	2	6	42	8	6	6	7	3	3
2013Garofolo_9263	3	Brucella_ITALIA_1	9263	Bufalo	Albanella,Italy	*B. abortus_biovar3*	Giuliano Garofolo		2011	36	72	MLVA16	4	5	3	12	2	2	3	1	6	42	8	5	6	7	3	3
2013Jiang#108	3	Brucella2013	NM1068	Cattle	Inner Mongolia, China	*B. abortus biovar 3*	Buyun Cui		1985	36		MLVA16	4	5	3	12	2	2	3	1	6	42	8	6	7	7	3	3
2013Jiang#105	3	Brucella2013	NM1065	Cattle	Inner Mongolia, China	*B. abortus biovar 3*	Buyun Cui		1985	117		MLVA16	4	5	3	12	2	2	3	2	6	42	8	6	4	4	3	3
2013Jiang#131	3	Brucella2013	NM1158	Cattle	Inner Mongolia, China	*B. abortus biovar 3*	Buyun Cui		1988	36		MLVA16	4	5	3	12	2	2	3	1	6	42	8	6	4	7	3	3
2013Jiang#140	3	Brucella2013	NM1175	Cattle	Inner Mongolia, China	*B. abortus biovar 3*	Buyun Cui		1990	36		MLVA16	4	5	3	12	2	2	3	1	6	42	8	6	4	7	3	3
2013Garofolo_3636	3	Brucella_ITALIA_1	3636	Cattle	Monte San Giacomo,Italy	*B. abortus_biovar3*	Giuliano Garofolo		2011	36	72	MLVA16	4	5	3	12	2	2	3	1	6	42	8	4	6	7	3	3
2013Garofolo_3916	3	Brucella_ITALIA_1	3916	Cattle	Monte San Giacomo,Italy	*B. abortus_biovar3*	Giuliano Garofolo		2011	36	72	MLVA16	4	5	3	12	2	2	3	1	6	42	8	4	6	7	3	3
2013Garofolo_3920	3	Brucella_ITALIA_1	3920	Cattle	Teggiano,Italy	*B. abortus_biovar3*	Giuliano Garofolo		2011	36	72	MLVA16	4	5	3	12	2	2	3	1	6	42	8	4	6	7	3	3
2013Garofolo_4363	3	Brucella_ITALIA_1	4363	Cattle	Laurenzana,Italy	*B. abortus_biovar3*	Giuliano Garofolo		2011	36	72	MLVA16	4	5	3	12	2	2	3	1	6	42	8	4	6	7	3	3
2013Garofolo_12183	3	Brucella_ITALIA_1	12183	Cattle	Corleto Monforte,Italy	*B. abortus_biovar3*	Giuliano Garofolo		2011	36	72	MLVA16	4	5	3	12	2	2	3	1	6	42	8	4	6	7	3	3
2013Garofolo_12185	3	Brucella_ITALIA_1	12185	Cattle	San Rufo,Italy	*B. abortus_biovar3*	Giuliano Garofolo		2011	36	72	MLVA16	4	5	3	12	2	2	3	1	6	42	8	4	6	7	3	3
2013Garofolo_21571	3	Brucella_ITALIA_1	21571	Cattle	Monte San Giacomo,Italy	*B. abortus_biovar3*	Giuliano Garofolo		2011	36	72	MLVA16	4	5	3	12	2	2	3	1	6	42	8	4	6	7	3	3
2013Garofolo_21675	3	Brucella_ITALIA_1	21675	Cattle	Monte San Giacomo,Italy	*B. abortus_biovar3*	Giuliano Garofolo		2011	36	72	MLVA16	4	5	3	12	2	2	3	1	6	42	8	4	6	7	3	3
2013Garofolo_22839	3	Brucella_ITALIA_1	22839	Bufalo	Albanella,Italy	*B. abortus_biovar3*	Giuliano Garofolo		2011	36	72	MLVA16	4	5	3	12	2	2	3	1	6	42	8	4	6	7	3	3
2013Garofolo_22842	3	Brucella_ITALIA_1	22842	Cattle	Teggiano,Italy	*B. abortus_biovar3*	Giuliano Garofolo		2011	36	72	MLVA16	4	5	3	12	2	2	3	1	6	42	8	4	6	7	3	3
2013Garofolo_5362	3	Brucella_ITALIA_1	5362	Bufalo	Monte San Giacomo,Italy	*B. abortus_biovar3*	Giuliano Garofolo		2011	36	72	MLVA16	4	5	3	12	2	2	3	1	6	42	8	4	6	7	3	3
2013Garofolo_8980	3	Brucella_ITALIA_1	8980	Cattle	Teggiano,Italy	*B. abortus_biovar3*	Giuliano Garofolo		2011	36	72	MLVA16	4	5	3	12	2	2	3	1	6	42	8	4	6	7	3	3
2013Garofolo_8984	3	Brucella_ITALIA_1	8984	Cattle	Sassano,Italy	*B. abortus_biovar3*	Giuliano Garofolo		2011	36	72	MLVA16	4	5	3	12	2	2	3	1	6	42	8	4	6	7	3	3
2013Jiang#093	3	Brucella2013	2011166	Human	Chongqing, China	*B. abortus biovar 3*	Buyun Cui		2011	36		MLVA16	4	5	3	12	2	2	3	1	6	42	8	4	6	7	3	3
2013Jiang#094	3	Brucella2013	YLQ	Human	Zhejiang, China	*B. abortus biovar 3*	Buyun Cui		2006	36		MLVA16	4	5	3	12	2	2	3	1	6	42	8	4	6	7	3	3
2013Jiang#104	4	Brucella2013	NM1061	Cattle	Inner Mongolia, China	*B. abortus biovar 3*	Buyun Cui		1984	112		MLVA16	4	5	3	12	2	2	3	3	6	42	8	5	4	7	3	3
2013Jiang#092	4	Brucella2013	2011165∖’	Human	Chongqing, China	*B. abortus biovar 3*	Buyun Cui		2011	36		MLVA16	4	5	3	12	2	2	3	1	6	42	8	4	7	7	3	3
2012Ferreira#146	4	Brucella2012	LNIV-328Ba3-06		Alentejo, Portugal	*B. abortus 3*	Cristina Ferreira	*B. abortus*	2006	36	72	MLVA16	4	5	3	12	2	2	3	1	6	42	8	4	7	7	3	3
2013Garofolo_3272	4	Brucella_ITALIA_1	3272	Cattle	Apricena,Italy	*B. abortus_biovar3*	Giuliano Garofolo		2011			MLVA16	4	5	3	12	2	2	3	3	6	42	8	7	6	3	3	3
2013Garofolo_18081	4	Brucella_ITALIA_1	18081	Cattle	Apricena,Italy	*B. abortus_biovar3*	Giuliano Garofolo		2011			MLVA16	4	5	3	12	2	2	3	3	6	42	8	7	6	3	3	3
2006LeFlèche#119	4	Brucella2012	BCCN#99-98	Cattle	Mongolia	*B. abortus 7*	Gilles Vergnaud	*B. abortus*	1999	36	72	MLVA16	4	5	3	12	2	2	3	1	6	42	8	5	6	4	3	3
2013Jiang#089	4	Brucella2013		Cattle	Hebei, China	*B. abortus biovar 3*	Buyun Cui		2011	36		MLVA16	4	5	3	12	2	2	3	1	6	42	8	4	5	7	3	3
2013Jiang#090	4	Brucella2013		Cattle	Hebei, China	*B. abortus biovar 3*	Buyun Cui		2011	36		MLVA16	4	5	3	12	2	2	3	1	6	42	8	4	5	7	3	3
2006LeFlèche#112	4	Brucella2012	BCCN#94-18	Cattle	Limoges, France	*B. abortus 3*	Gilles Vergnaud	*B. abortus*	1994	36	72	MLVA16	4	5	3	12	2	2	3	1	6	42	8	4	5	7	3	3
2013Jiang#100	4	Brucella2013	NM1051	Cattle	Inner Mongolia, China	*B. abortus biovar 3*	Buyun Cui		1984	36		MLVA16	4	5	3	12	2	2	3	1	6	42	8	6	4	8	3	3
2006LeFlèche#005	4	Brucella2012	REF 292	Cattle	England	*B. abortus 4*	Gilles Vergnaud	*B. abortus*		30	78	MLVA16	4	5	4	12	2	2	3	2	6	42	8	3	4	3	3	5
2009Her#004	4	Brucella2012	KRef04	Cattle	England	*B. abortus 4*	Moon Her	*B. abortus*		30	78	MLVA16	4	5	4	12	2	2	3	2	6	42	8	3	4	3	3	5
2012Ferreira#213	4	Brucella2012	REF 292			*B. abortus 4*	Cristina Ferreira	*B. abortus*		30	78	MLVA16	4	5	4	12	2	2	3	2	6	42	8	3	4	3	3	5
2013Jiang#127	4	Brucella2013	NM1147	Cattle	Inner Mongolia, China	*B. abortus biovar 3*	Buyun Cui		1988	117		MLVA16	4	5	3	12	2	2	3	2	6	42	8	5	4	3	3	3
2013Jiang#130	4	Brucella2013	NM1156	Sheep	Inner Mongolia, China	*B. abortus biovar 3*	Buyun Cui		1988	36		MLVA16	4	5	3	12	2	2	3	1	6	42	8	6	4	4	3	3
2013Jiang#125	4	Brucella2013	NM1140	Cattle	Inner Mongolia, China	*B. abortus biovar 3*	Buyun Cui		1988	36		MLVA16	4	5	3	12	2	2	3	1	6	42	8	5	4	7	3	3
2013Jiang#126	4	Brucella2013	NM1146	Cattle	Inner Mongolia, China	*B. abortus biovar 3*	Buyun Cui		1988	36		MLVA16	4	5	3	12	2	2	3	1	6	42	8	5	4	7	3	3
2013Jiang#128	4	Brucella2013	NM1148	Cattle	Inner Mongolia, China	*B. abortus biovar 3*	Buyun Cui		1988	36		MLVA16	4	5	3	12	2	2	3	1	6	42	8	5	4	7	3	3
2013Jiang#141	4	Brucella2013	NM1176	Cattle	Inner Mongolia, China	*B. abortus biovar 3*	Buyun Cui		1990	36		MLVA16	4	5	3	12	2	2	3	1	6	42	8	5	4	7	3	3
2013Jiang#150	4	Brucella2013	NM1215	Cattle	Inner Mongolia, China	*B. abortus biovar 3*	Buyun Cui		1994	36		MLVA16	4	5	3	12	2	2	3	1	6	42	8	5	4	7	3	3
2013Jiang#151	4	Brucella2013	NM1218	Cattle	Inner Mongolia, China	*B. abortus biovar 3*	Buyun Cui		1995	36		MLVA16	4	5	3	12	2	2	3	1	6	42	8	5	4	7	3	3
2013Jiang#146	4	Brucella2013	NM1185	Cattle	Inner Mongolia, China	*B. abortus biovar 3*	Buyun Cui		1990	36		MLVA16	4	5	3	12	2	2	3	1	6	42	8	6	4	5	3	3
2013Jiang#152	4	Brucella2013	NM1219	Cattle	Inner Mongolia, China	*B. abortus biovar 3*	Buyun Cui		1995	36		MLVA16	4	5	3	12	2	2	3	1	6	42	8	5	5	7	3	3
2013Jiang#113	4	Brucella2013	NM1075	Cattle	Inner Mongolia, China	*B. abortus biovar 3*	Buyun Cui		1985	117		MLVA16	4	5	3	12	2	2	3	2	8	42	8	5	6	3	3	3
2006LeFlèche#135	4	Brucella2012	BfR 95	Mouse	?	*B. abortus 1*	Gilles Vergnaud	*B. abortus*		28	82	MLVA16	4	5	4	12	2	2	3	3	6	42	8	3	6	3	3	5
2009Her#011	4	Brucella2012	KRef15	Cattle	USA	*B. abortus 1*	Moon Her	*B. abortus*		28	82	MLVA16	4	5	4	12	2	2	3	3	6	42	8	3	6	3	3	5
2013Jiang#083	4	Brucella2013		Cattle	Xinjiang, China	*B. abortus biovar 3*	Buyun Cui		2011	36		MLVA16	4	5	3	12	2	2	3	1	6	42	8	4	6	5	3	3
2013Garofolo_3921	4	Brucella_ITALIA_1	3921	Cattle	San Gregorio Magno,Italy	*B. abortus_biovar3*	Giuliano Garofolo		2011	36	72	MLVA16	4	5	3	12	2	2	3	1	6	42	8	4	6	6	3	3
2013Garofolo_5007	4	Brucella_ITALIA_1	5007	Cattle	San Gregorio Magno,Italy	*B. abortus_biovar3*	Giuliano Garofolo		2011	36	72	MLVA16	4	5	3	12	2	2	3	1	6	42	8	4	6	6	3	3


Finally, based on these typing results, strain 8416 might be a new variant of *B. abortus* biovar 9.

## Discussion

Until now, the phage resistance mechanism from *Brucella* SPR strains was poorly understood. In this study, a natural SPR strain of *B. abortus* isolated from a patient in China was identified. Although SPR strains of *B. abortus* were rarely isolated from patients, a SPR strain was isolated from a *B. abortus* phage sensitive parent strain 544 in 1974 and a SPR variant of *B. abortus* strain 19 was identified in 1976 through the manipulation of laboratory cultures (Corbel and Morris, 1974; Corbel and Thomas, 1976). Compared to the parent strain 544, the SPR strain FS showed no differences in virulence, morphological, cultural, biochemical or metabolic, and serological reactions, but with an altered phage resistance profile (Corbel and Morris, 1974). The potential mechanism of the phage resistance may be due to its failure to penetrate the FS cell wall since the strain FS is more resistant to lysis by phage lysozymes than that of the phage-sensitive parent strain 544 (Corbel and Morris, 1975). Strain 544-FS showed a complete resistance to lysis by many *Brucella* phages except Bk_2_ at 1× RTD and 10^4^× RTD. Subsequently, another *B. abortus* SPR strain with resistance to phage Tb, was isolated from a supramammary lymph node of a cow and it is virulent to guinea-pigs (Harrington et al., 1977). Interestingly, these *B. abortus* SPR strains mentioned above belonging to *B. abortus* biovar 1 were identified. However, strain 8416 was significantly different from all of *B. abortus* biovars by using phenotypic and molecular typing method. However, it shared the same phage lysis profiles to that of *B. melitensis* biovar 1. In conclusion, strain 8416 is the only SPR strain isolated from the infected human thus far with a similar phage lysis pattern with *B. melitensis* 16 M. However, despite same resistant to phage Tb, we could not comprehensively compare with phage lysis profiles of the three reported SPR strains due to different *Brucella* phages tested among them.

Currently, MLVA has been mainly used for tracking the variances of the bacterial genus with a high homology, such as *Brucella* genus (Haguenoer et al., 2011). The MLVA-16 (panel 1, 2A and 2B) assay was widely used for molecular typing of a larger collection of isolates at both species and biovars level. The panel 1 comprised eight minisatellite markers for species identification (Le Fleche et al., 2006) and the panel 2 markers were found with a higher biovar discriminatory power. Surprisingly, the MLVA-16 typing results showed that strain 8416 was clustered into the Chinese *B. abortus* biovar 3 strains (Jiang et al., 2013) with four variable loci (bruce04, 07, 11, and 55). Actually, among the four known panel 1 genotypes (28, 30, 112, 116), strain 8416 (genotype 30) was distinct from other 65 Chinese *B. abortus* biovar 3 strains isolated previously from different geographic origins, suggesting that more *B. abortus* strains phenotypically identified as biovar 3 are required for the comparison. The MLVA assay confirmed that *B. abortus* biovar 3 is a heterogeneous group (Le Fleche et al., 2006), and in agreement with the *B. abortus* biovar 3 divided into two sub-biovar 3a and 3b (Huber et al., 2009).

In this study, an atypical *B. abortus* strain displaying a phage lysis profile similar to *B. melitensis* biovars 1 was identified. Most importantly, the lysis pattern by bacteriophages observed in this newly uncovered *B. abortus* SPR strain. Although phage typing in general can successfully classify *Brucella* species, our research calls for attention as to conclusions on SPR strains. Further investigation focusing on the strain 8416’s whole genomic variations associated with phage resistance is needed.

## Conflict of Interest Statement

The authors declare that the research was conducted in the absence of any commercial or financial relationships that could be construed as a potential conflict of interest.
